# Lipoprotein Lipase Inhibits Hepatitis C Virus (HCV) Infection by Blocking Virus Cell Entry

**DOI:** 10.1371/journal.pone.0026637

**Published:** 2011-10-21

**Authors:** Patrick Maillard, Marine Walic, Philip Meuleman, Farzin Roohvand, Thierry Huby, Wilfried Le Goff, Geert Leroux-Roels, Eve-Isabelle Pécheur, Agata Budkowska

**Affiliations:** 1 Institut Pasteur, Unité Hépacivirus et Immunité Innée, Département de Virologie, Paris, France; 2 CNRS, URA3015, Paris, France; 3 Univ. Paris Diderot, Sorbonne Paris Cité, Cellule Pasteur, Paris, France; 4 Center for Vaccinology, Ghent University and Hospital, Ghent, Belgium; 5 Pasteur Institute of Iran, Hepatitis and AIDS Department, Teheran, Iran; 6 INSERM, UMRS939, Dyslipidemia, Inflammation and Atherosclerosis in Metabolic Diseases, Paris, France; 7 Université Lyon 1, Univ. Lyon, Lyon, France; 8 CNRS, UMR5086, Lyon, France; 9 IBCP, Bases Moléculaires et Structurales des Systèmes Infectieux, Lyon, France; Nanyang Technological University, Singapore

## Abstract

A distinctive feature of HCV is that its life cycle depends on lipoprotein metabolism. Viral morphogenesis and secretion follow the very low-density lipoprotein (VLDL) biogenesis pathway and, consequently, infectious HCV in the serum is associated with triglyceride-rich lipoproteins (TRL). Lipoprotein lipase (LPL) hydrolyzes TRL within chylomicrons and VLDL but, independently of its catalytic activity, it has a bridging activity, mediating the hepatic uptake of chylomicrons and VLDL remnants. We previously showed that exogenously added LPL increases HCV binding to hepatoma cells by acting as a bridge between virus-associated lipoproteins and cell surface heparan sulfate, while simultaneously decreasing infection levels. We show here that LPL efficiently inhibits cell infection with two HCV strains produced in hepatoma cells or in primary human hepatocytes transplanted into uPA-SCID mice with fully functional human ApoB-lipoprotein profiles. Viruses produced *in vitro* or *in vivo* were separated on iodixanol gradients into low and higher density populations, and the infection of Huh 7.5 cells by both virus populations was inhibited by LPL. The effect of LPL depended on its enzymatic activity. However, the lipase inhibitor tetrahydrolipstatin restored only a minor part of HCV infectivity, suggesting an important role of the LPL bridging function in the inhibition of infection. We followed HCV cell entry by immunoelectron microscopy with anti-envelope and anti-core antibodies. These analyses demonstrated the internalization of virus particles into hepatoma cells and their presence in intracellular vesicles and associated with lipid droplets. In the presence of LPL, HCV was retained at the cell surface. We conclude that LPL efficiently inhibits HCV infection by acting on TRL associated with HCV particles through mechanisms involving its lipolytic function, but mostly its bridging function. These mechanisms lead to immobilization of the virus at the cell surface. HCV-associated lipoproteins may therefore be a promising target for the development of new therapeutic approaches.

## Introduction

HCV hepatitis C virus (HCV) infection is a major cause of liver disease worldwide. In most cases, HCV infection progresses to chronic liver disease, which can lead to liver cirrhosis and hepatocarcinoma [1]. There is still no vaccine available, and current therapies have only limited efficacy, depending on the virus genotype, and are associated with several side effects [2], [3].

HCV is an enveloped virus of the *Flaviviridae* family (genus *Hepacivirus*) with a single-stranded positive RNA genome of about 9.6 kb. The viral genome encodes a polyprotein composed of about 3,000 amino acids, which is cleaved by the host and viral proteases into three structural proteins (the capsid protein, and two envelope glycoproteins E1 and E2), P7 and several nonstructural proteins (NS2, NS3, NS4A, NS4B, NS5A and NS5B). The structural proteins form the virus particles, whereas nonstructural proteins NS3 to NS5 are involved in genome replication and virus assembly (reviewed in [4]).

A distinctive feature of the HCV life-cycle is that viral RNA replication depends on cholesterol and fatty acid biosynthesis [5], [6], [7]. In addition, the assembly and secretion of viral progeny are closely related to the VLDL biogenesis pathway [5], [7], [8], [9]. Chronic HCV infection thus induces changes in host lipid metabolism, such as a decrease in serum lipoprotein levels and an accumulation of lipids in liver parenchymal cells (steatosis) [10], [11], [12].

HCV particles contain the nucleocapsid and the envelope E1 and E2 glycoproteins. Nevertheless, most of the HCV in the serum of patients is of low density [13], [14] and has a VLDL-like structure. Indeed, it has been suggested that HCV is a hybrid particle composed of TRL containing ApoB and ApoE, in which the HCV nucleocapsids and virus envelope glycoproteins are embedded [15], [16]. These viral structures, known as “Lipo Viro Particles” (LVPs), constitute the most infectious population of HCV, as shown by studies in a chimpanzee [14] and in cell culture models [15], [17]. The association of cholesterol and sphingolipids with HCV is essential for virus maturation and infectivity [18], [19].

Due to the crucial role of lipoproteins in the virus cell cycle, there is increasing evidence that a powerful tool for studying HCV infection — a cell culture–derived HCV (HCVcc) allowing *in vitro* replication and the production of infectious virus particles — does not accurately reflect the real infection process in terms of the lipoprotein composition of the virus and host cell phenotype. Indeed, comparative analyses of the viruses produced in hepatoma cells and in experimental models showed that the HCV produced *in vivo* had a lower density and a higher specific infectivity [20] and fusogenic capacity [21] than HCVcc produced *in vitro*.

The data currently available suggest that lipoproteins integrated into viral particles play an important role in virus uptake into the hepatocyte (for review, see [22]). The initiation of a productive HCV infection involves several cellular factors that trigger virus uptake via tight junctions, and three of the major molecules mediating HCV cell entry, the scavenger receptor (SR-BI), the LDL-receptor (LDL-R) and heparan sulfate proteoglycans (HSPG) also interact with lipoproteins or lipids.

The essential role of lipoproteins in the HCV life cycle led us to investigate the role of lipoprotein lipase in viral uptake and HCV infection. LPL is a lipolytic enzyme (EC 3.1.1.34) that hydrolyzes lipids in TRL present in chylomicrons and VLDL [23]. LPL is produced by muscle and adipose tissue, but is specifically found attached to the endothelial cells lining capillaries on the luminal side, where it catalyzes the lipolysis of ApoB- and ApoE-containing lipoproteins. Hydrolysis of the triacylglycerol component of circulating TRL provides fatty acids for peripheral tissues, which are internalized via CD-36 [24], [25], [26].

In addition to its catalytic function as a triglyceride hydrolase, the homodimeric form of LPL has a second, “structural” function, as a bridging factor for lipoprotein uptake by the hepatocyte, probably leading to the cellular catabolism of lipoproteins [26], [27]. This function of LPL mediates the hepatic clearance of VLDL and chylomicron remnants independent of the lipolytic function of the enzyme: indeed, enhanced lipoprotein uptake is not affected by inhibitors of its lipolytic activity in either *in vitro* or *in vivo* models [24], [27], [28]. The enzyme promotes the hepatic uptake of lipoproteins via liver HSPG (syndecan-1) alone [26], [29], [30] or HSPG interacting with lipoprotein receptors: LDL-R, LRP [25], [28] or SR-BI [31].

In a previous study, we showed that LPL enhances the binding of HCV from the sera of patients to various cell types, including hepatoma cell lines [32]. Like the mechanisms operating for lipoproteins, the mechanisms of action of LPL on HCV involved the formation of a bridge by the dimeric form of LPL between virus-associated lipoproteins and cell-surface HSPG. Our previous observations also showed an inhibitory effect of LPL on HCV infection in the HCV cell culture model (HCVcc) [32].

This intriguing observation led us to investigate further the influence of LPL on cell infection by HCV. We first compared the effect of LPL on cell infection with different HCV strains produced *in vitro* in hepatoma cells (which have defective lipoprotein metabolism) with its effect on cell infection by the virus produced in primary human hepatocytes transplanted into uPA/SCID mice, a model mimicking the natural infection of differentiated human hepatocytes with normal lipid and lipoprotein metabolism [33], [34], [35], [36]. We then analyzed the mechanism of action of LPL on HCV infection, which involves the LPL catalytic function, but is dependent mostly on the structural function of the enzyme. Our immunoelectron microscopy studies showed that LPL inhibits HCV cell entry, blocking the virus at the cell surface.

## Materials and Methods

### Cell culture

Human Huh7.5 hepatoma cells (kindly provided by C. Rice) were grown in Dulbecco's modified Eagle's medium (DMEM; Invitrogen, Cergy Pontoise, France) supplemented with sodium pyruvate, 10% fetal calf serum, glutamine, antibiotics, antifungal agents and non essential amino acids. Cells were maintained at 37°C, under an atmosphere containing 5% CO_2_.

### Virus strains

The plasmid encoding the genome of the JFH-1 strain was used to generate HCVcc. The virus was cultured as previously described [37], to obtain a viral stock of 10^7^ IU/ml.

The pFI-J6J plasmid, used to generate the J6/JFH-1 virus strain, was kindly provided by C. Rice. JFH-1/J6 HCV genomic RNA was obtained from purified pFI-J6J with the T7 Ribomax Express large-scale RNA production system (Promega). Huh7.5 cells were transfected and cultured as previously described [38], to obtain a viral stock of 7.9×10^6^ IU/ml.

### Cell infection with HCV in the presence and absence of LPL

Confluent monolayers of Huh7.5 cells were grown for 48 h in six-well tissue culture plates to obtain approximately 10^6^ cells/well. Cells were incubated with 25 µl of the virus preparations for 2 h at 37°C to allow infection. Cells were washed and grown in complete DMEM for 24 h or 48 h at 37°C. Total RNA was then extracted and HCV RNA levels were determined by quantitative RT-PCR. Data were normalized with respect to the cellular gene GAPDH, using the GAPDH Control Kit (Eurogentec, Angers, France). All assays were performed at least in triplicate. HCV RNA levels are expressed in IU relative to a HCV RNA quantification panel from Acrometrix (Berkeley, CA, USA).

We assessed the effect of lipoprotein lipase on HCV infection, by incubating Huh7.5 cells with 1 µg/ml bovine LPL (Sigma) diluted in complete DMEM for 30 min at 4°C before adding the virus.

For investigation of the enzymatic effect of LPL, Huh7.5 cells were incubated for 30 min at 4°C with 1 µg/ml LPL diluted in complete DMEM in the presence of a lipase inhibitor, tetrahydrolipstatin (THL, Calbiochem, Darmstadt, Germany) at a final concentration of 50 µg/ml, before adding the virus. Inoculation was carried out for 2 h at 37°C. Cells were then washed and grown in complete DMEM for 24 h or 48 h. Total RNA was extracted and HCV RNA levels were determined by quantitative RT-PCR. Data were normalized with respect to GAPDH, as described above.

### Quantitative RT-PCR (RT-qPCR)

The HCV RNA associated with cells was quantified by one-step quantitative RT-PCR with the SuperScript III Platinum One-Step qRT-PCR Kit (Invitrogen). The 5′-AGYGTTGGGTYGCGAAAG-3′ and 5′-CACTCGCAAGCRCCCT-3′ primers were used to amplify the HCV RNA from JFH clones, and 6-FAM-CCTTGTGGTACTGCCTGA-MGB (Applied Biosystems, Foster City, CA, USA) was used as an internal probe. Real-time detection of the PCR products was carried out with an AbiPrism 7000. HCV RNA was quantified relative to a reference viral stock and standardized with an HCV RNA quantification panel from AcroMetrix. RNA levels are expressed as HCV RNA IU.

### Infection of chimeric uPA-SCID mice with JFH-1 and J6/JFH-1 HCVcc

uPA-SCID mice with engrafted human liver cells were generated as previously described [33], [34]. Briefly, 10^6^ cryopreserved primary human hepatocytes (BD Biosciences, Erembodegem, Belgium) were transplanted into two-week old uPA^+/+^-SCID mice. Two months later, the concentration of human albumin in mouse plasma was quantified, to assess the numbers of human hepatocytes engrafted into the mouse liver (Bethyl Laboratories Inc., Montgomery, TX, USA). Chimeric mice were infected with a virus stock prepared from a concentrated cell culture supernatant containing JFH-1 at a final concentration of 10^9^ IU/ml and/or J6/JFH-1 at a concentration of 7.9×10^6^ IU/ml.

The JFH-1 preparation produced in mice (mJFH-1) was generated by pooling all plasma samples collected between 1 and 12 weeks after inoculation. A pool of plasma from J6/JFH-1-infected mice (mJ6/JFH-1) was obtained by combining samples collected between days 7 and 38 post-infection. The viral titers of the mJFH-1 and mJ6/JFH-1 pools were 5.85×10^5^ IU/ml and 7.82×10^7^ IU/ml, respectively, as determined with the Roche COBAS Ampliprep – COBAS TaqMan HCV test (Roche Diagnostics, Vilvoorde, Belgium).

### Ethics Statement

This study was carried out in strict accordance with the recommendations for the care and use of the laboratory animals of the European Community (Directive 86/609/EEC). The protocol was approved by the Animal Ethics Committee of the Faculty of Medicine and Health Sciences, Ghent University, Belgium (Permit Number EDC06/07). During surgery, the animals were anesthetized with isoflurane and all possible efforts were made to minimize suffering.

### Ultracentrifugation through iodixanol gradients

Discontinuous 5-50% iodixanol density gradients (OptiPrep, Sigma) were prepared from five buffered solutions of iodixanol in HEPES-buffered saline (Sigma). Samples were overlaid onto the gradients and centrifuged for 22 h at 4°C and 50,000 rpm in a SW60Ti rotor in a Beckman Coulter Optima L-90 K ultracentrifuge. Fractions (50 µl each) were collected and their density was determined with a refractometer (Atago CO LTD, Tokyo, Japan).

### Determination of HCV infectivity

We determined the infectivity of the viral preparation, by infecting Huh7.5 cells (10^5^ per well in a 24-well plate) with 25 µl of each virus fraction and incubating for 48 h at 37°C. Total RNA was extracted and HCV-RNA levels were quantified by quantitative RT-PCR. The results were normalized, taking into account the initial HCV-RNA content of each of the samples analyzed by RT-qPCR.

### Determination of cellular lipid mass in the presence and absence of LPL

Huh7.5 cells (0.2×10^6^ cells/well) were used to seed 24-well plates, which were incubated for 24 h at 37°C. Cells were then washed with cold PBS and incubated for 5 min on ice with cold serum-free medium before adding 5 µg/ml bovine LPL (Sigma), with or without 50 µg/ml LPL inhibitor, tetrahydrolipstatin (THL), (Sigma). After incubation on ice for 20 minutes, 50 µg/ml human VLDL-Prot (d<1.006 g/ml) isolated from normolipidemic plasma by preparative ultracentrifugation [39] was added to the culture medium, which was then incubated for an additional 30 minutes on ice. When required, cells were incubated with 10 U/ml heparin (Choay) for 10 min on ice and then for 2 hours at 37°C. Total cellular triglycerides and cholesterol mass were quantified as previously described [40].

### Electron microscopy

Supernatants from Huh7.5 cells producing JFH1 HCVcc were concentrated by centrifugation through a 20% sucrose cushion for 4 h at 4°C and 32,000 rpm, in a SW32Ti rotor.

Huh7.5 cells were grown in 3 cm dishes for 24 h, washed with cold serum-free DMEM and maintained for 10 min at 4°C before replacing the medium with 1 ml cold serum with or without DMEM and 1 µg of LPL, and incubating for a further 20 min at 4°C. Concentrated virus preparation (containing 5×10^9^ IU/ml of HCV RNA) was then added and incubated with cells for 30 min at 4°C. At the end of this adsorption step at 4°C, cells were either washed immediately (T0) or placed at 37°C for 5 min (T5), 10 min (T10) or 20 min (T20). Cells were then prepared for transmission electron microscopy (TEM) examination. They were lightly fixed by incubation with 4% (v/v) paraformaldehyde (Delta Microscopies, Ayguevive, France) and 0.05% (v/v) glutaraldehyde in phosphate-buffered saline (PBS) at pH 7.4, for 30 min at 37°C. They were then thoroughly washed with PBS and incubated for 10 min in blocking solution composed of 0.5% v/v cold fish skin gelatin (FSG), 0.1% v/v saponin and 0.02 M glycine (all from Sigma Aldrich) in PBS pH 7.4, at room temperature. For control experiments, saponin-free buffer was used. After washing with 0.5% FSG in PBS (PBS/FSG), cells were incubated for 60 min with primary monoclonal antibodies diluted in PBS/FSG: anti-E2 AP-33, (diluted 1∶1500), kindly provided by A. Patel, or ACAP-27 (0.6 µg/ml), kindly provided by JF. Delagneau, or anti-LPL (Sigma, at a concentration of 1.66 µg/ml). After washing in PBS/FSG, cells were then incubated for 30 min with secondary, anti-mouse IgG antibody conjugated with Nanogold® particles (Yaphank, NY, USA) in PBS/FSG (1∶75), washed extensively in PBS/FSG and fixed by incubation with 1% v/v glutaraldehyde in PBS, for 1 h, at room temperature. Silver enhancement of Nanogold® was carried out for 3 min, after washing in distilled water, with the HQ silver enhancement kit (Nanoprobe, Yaphank, NY, USA) according to the manufacturer's instructions. Cells were then post-fixed by incubation with 1% v/v osmium tetroxide in PBS for 1 h and processed by dehydration in ethanol for embedding in epoxy resin. TEM examination was performed on a Philips CM120 microscope operating at 80 kV, and images were acquired with an ORIUS SC200D CCD camera (Gatan Inc.).

HCV-bound gold particles were quantified in LPL-treated and non treated preparations, for each time point. We examined 30 cells in each case and determined the number of intra- or extracellular gold particles.

### Other assays

HCV core antigen was determined in gradient fractions with the HCV Core Antigen ELISA kit (WAKO Chemicals, Ortho-Clinical Diagnostics, Germany). Apolipoprotein B was assayed with the AssayMax Human Apolipoprotein B ELISA kit (AssayPro, St. Charles, MO, USA) and cholesterol was quantified with the Amplex® Red Cholesterol Assay Kit (Invitrogen).

## Results

### LPL inhibits infection with two HCVcc strains produced in Huh7.5 cells

As hepatic cells do not produce LPL, we used bovine LPL to investigate the effect of exogenously added enzyme on virus multiplication in Huh7.5 cells. This cell line is capable of supporting the full HCV cell cycle. Bovine LPL is very similar to human LPL and is thus often used in studies on the role of LPL in interactions of lipoproteins with cells [41]. Our previous experiments suggested that LPL inhibited cell infection with HCVcc (JFH-1 strain), with a large decrease in virus production observed 24 h, 48 h and 72 h post-infection [32]. We extended this observation, using another virus strain, J6/JFH-1, which also replicates in Huh7.5 cells. The JFH-1 strain is based on a cloned genotype 2a HCV RNA isolated from a patient with fulminant hepatitis C [37], whereas, in the chimeric J6/JFH-1 strain, the genes encoding the structural (E1,E2,core), p7 and NS2 proteins are derived from another virus, of genotype 2a (HC-J6) [38].

**Figure 1:**
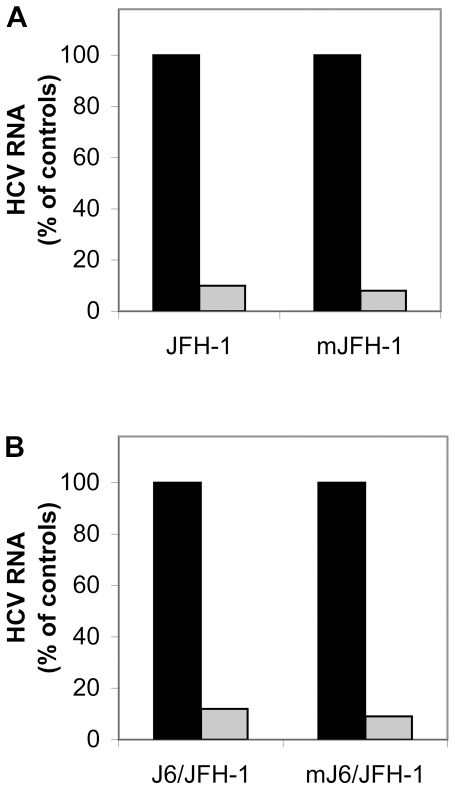
LPL inhibits cell infection by the JFH-1 and J6/JFH-1 strains produced *in vitro* and *in vivo* in a chimeric uPA-SCID mouse model. The HCVcc strains JFH-1 (A) and J6/JFH-1 (B) were produced in the Huh7.5 hepatoma cell line. Cells were incubated with (or without) LPL for 30 min at 4°C and then with virus preparations for 2 h at 37°C to allow infection. RNA was extracted from cells 24 h post infection and HCV RNA was quantified by RT-qPCR. The data obtained were normalized with respect to levels of GADPH. The mJFH-1 (A) and mJ6/JFH-1 (B) correspond to HCVcc strains produced in chimeric uPA-SCID mice into which we transplanted human hepatocytes. Serum samples collected from infected mice were pooled and their capacity to infect Huh7.5 cells was assessed in the presence and absence of LPL, as outlined above. Cells infected in the absence (black bar) and in the presence of LPL (gray bar). The data are expressed as the amount of HCV RNA detected in cells infected in the presence of LPL as compared with the amount of HCV RNA in cells infected in the absence of LPL, expressed as a percentage.

We investigated the effect of LPL on cell infection with these two HCVcc strains, by incubating Huh7.5 cells with bovine LPL for 30 min at 4°C before adding the virus, and determining intracellular HCV RNA levels 24 h after infection, as previously described [32]. The infection of cells with the two virus strains was inhibited by more than 90% in the presence of LPL (Figure 1A). This inhibition was also observed 48 and 72 h after infection. Thus, the addition of LPL to Huh7.5 cells as an exogenous ligand inhibited cell infection with various HCVcc strains.

### LPL inhibits cell infection with HCV produced in primary human hepatocytes transplanted into chimeric uPA-SCID mice

Lipids and lipoproteins are required for HCV replication, and for the morphogenesis and secretion of infectious virus particles [5], [6], [7], [42]. The Huh7.5 cell line commonly used to propagate HCV in cell culture differs in several ways, in terms of its lipoprotein metabolism, from human primary hepatocytes [42], [43], [44], [45]. Indeed, this cell line secretes relatively dense, lipid-poor ApoB-containing lipoprotein particles, unlike the VLDL produced *in vivo* by the human liver. Uncertainties therefore remain concerning the extent to which the HCVcc produced in these cells resembles real, lipoprotein associated HCV. It was therefore important to determine whether our observations with HCVcc produced in cell culture were relevant to virus produced in primary human hepatocytes.

We investigated whether LPL inhibited cell infection with the same virus strains produced in primary human hepatocytes transplanted into chimeric mice. In this model, severe combined immunodeficiency disorder (SCID) mice carry a urokinase-type plasminogen activator transgene controlled by an albumin promoter (Alb-uPA), inducing a hepatocyte-lethal phenotype. It is therefore possible to transplant primary human hepatocytes into uPA-SCID mice. Repopulation occurs in a well organized fashion and the mice display human-type liver metabolism and can be infected with HCV of various genotypes [33], [34]. Importantly, the viral particles produced in these mice have characteristics similar to those of viruses isolated from HCV-infected chimpanzees and patients with chronic HCV infection [20] .

The JFH-1 and J6/JFH-1 HCVcc strains produced in Huh7.5 cells were concentrated and injected into chimeric uPA-SCID mice, to infect transplanted human primary hepatocytes. We assessed the ability of pooled serum samples from infected mice (designated mJFH-1 and mJ6/JFH-1) to infect Huh7.5 cells in the presence or absence of LPL. Cell infection with these two virus strains produced in chimeric mice was strongly inhibited in the presence of LPL, like infection with viruses produced in Huh7.5 cells (Figure 1 A,B).

Thus, exogenously added LPL has a potent inhibitory effect on cell infection with HCV produced in hepatoma cells *in vitro* or *in vivo* in human primary hepatocytes transplanted into chimeric mice.

### Analysis of viruses produced *in vitro* and *in vivo*

HCV particles produced *in vitro* [20], [46] and *in vivo* [16], [20], [46] are heterogeneous and may have different buoyant densities due to their association with different classes of lipoproteins. To determine the density profiles of the virus particles generated in cell culture and in humanized mice and to identify the virus fraction sensitive to LPL, we analyzed these virus preparations fractionated on iodixanol gradients. This type of gradient is frequently used for the analysis of lipoproteins and has been used to separate HCV-lipoprotein complexes from the serum of patients [16].

Concentrated supernatants from Huh7.5 cells were subjected to isopycnic centrifugation through an iodixanol gradient. The fractions collected were analyzed for the presence of HCV core antigen, HCV RNA, ApoB and cholesterol.

**Figure 2:**
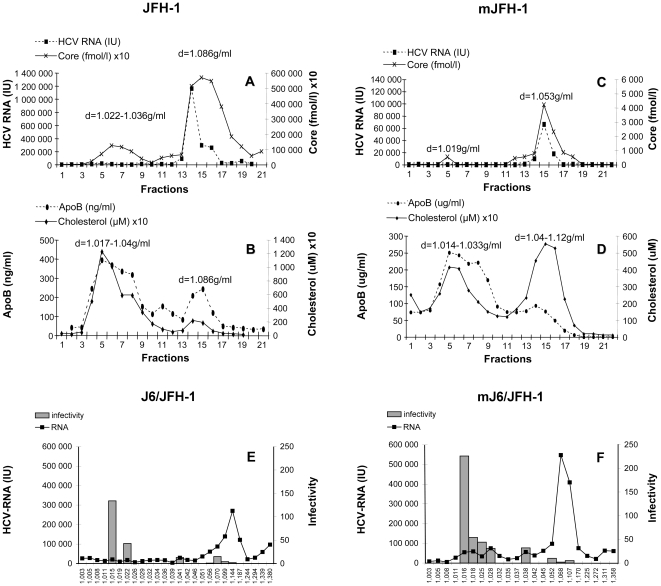
Iodixanol gradient analysis of the JFH-1 and J6/JFH-1 strains produced *in vitro* and *in vivo*. The supernatants from infected Huh7.5 cells producing JFH-1 (JFH-1, shown in A and B) and J6/JFH-1 (shown in E) were subjected to isopycnic centrifugation through iodixanol gradients, as described in Materials and Methods. Pooled serum samples from the chimeric uPA-SCID mice were also subjected to centrifugation on the same type of gradient. Representative profiles are shown in C and D for mice inoculated with JFH-1 (mJFH-1) and in F for mice inoculated with J6/JFH-1 (mJ6/JFH-1). HCV core antigen in gradient fractions was quantified by ELISA, HCV RNA was quantified by RT-qPCR, and ApoB and cholesterol were determined by ELISA. Infectivity for fractionated J6/JFH-1 (representative for both strains) grown in Huh7.5 cells is shown in E and that for the corresponding mouse serum (mJ6/JFH-1) is shown in F. The fractions (25 µl) were used to infect Huh7.5 cells. Cells were incubated for 48 h at 37°C; total RNA was then extracted and HCV-RNA levels were quantified by RT-qPCR. The results were normalized, taking into account the initial HCV-RNA content in each sample analyzed, as determined by RT-qPCR, and are expressed as a ratio of these two values.

For the JFH-1 strain, HCV-RNA was detected at various concentrations in most fractions of the gradient with a peak at a density of 1.066 g/ml. HCV core antigen was found mostly in two peaks, delineating two viral populations (Figure 2A). The first of these populations banded in the density range of 1.022–1.036 g/ml and colocalized with most of the Apo B and cholesterol (detected in the density range 1.017–1.04 g/ml) (Figure 2B). The second viral peak, containing higher levels of HCV core antigen, partly coincided with HCV RNA and was recovered at a mean density of 1.086 g/ml. ApoB and cholesterol were also found at this density, but in lower concentrations than in the lower-density virus population. A similar distribution of the viral RNA was obtained for the J6/JFH-1strain (Figure 2E). For both virus strains, fractions corresponding to the main HCV RNA peak were of relatively low infectivity (Figure 2F). Thus, there was probably a large excess of RNA over infectious virus particles in this peak. Indeed, the high density population was found to be about 8 to 10 times less infectious than the low-density viral peak. These findings confirm previous observations of an inverse relationship between virus density and infectivity [37], [38], [46], [47].

The human hepatoma cell line Huh7.5 does not display typical lipoprotein metabolism. The virus particles produced in this *in vitro* model may therefore have lipoprotein compositions and physicochemical properties different from those of virus particles produced in primary hepatocytes. As the association of HCV in the blood of chimeric mice with ApoB-containing lipoproteins is similar to that observed in the serum of patients infected with HCV [36], the properties of virus particles produced in this model may more closely mimic those of particles recovered from the blood of HCV-infected patients.

We compared the properties of virus particles produced *in vitro* and *in vivo* by analyzing sera from HCV-infected mice on iodixanol gradients. HCV from infected chimeric mice, such as the cell culture-derived HCVcc, banded mainly in two peaks in the iodixanol gradients. For the JFH-1 strain recovered from mice (mJFH-1), a low–density virus population appeared as a very small peak of HCV-core antigen, banding at a density of 1.019 g/ml, whereas a higher density mJFH-1 population was recovered as a peak containing both HCV RNA and core antigen at a density of 1.053 g/ml (Figure 2C). Both viral peaks colocalized with cholesterol and ApoB, although most of the ApoB was associated with the low-density population (Figure 2D). Low density HCVcc population produced in mice was 15 times more infectious than higher density population from the same gradient (Figure 2F).

When compared viruses produced *in vivo* and *in vitro* the first, low density virus population identified in fractionated mice sera was about twice as infectious as the corresponding population from the inoculum (Fig. 2F). The second, higher density virus peak in fractionated mice serum containing most of the HCV RNA and core antigen and had a significantly lower buoyant density than the corresponding peak from the initial inoculum (1.053 versus 1.066 g/ml and 1.06 versus and 1.14 g/ml, for JFH-1 and J6/JFH-1, respectively). These changes in viral density corresponded to an increase in infectivity by a factor of about three (Figure 2F).

**Figure 3:**
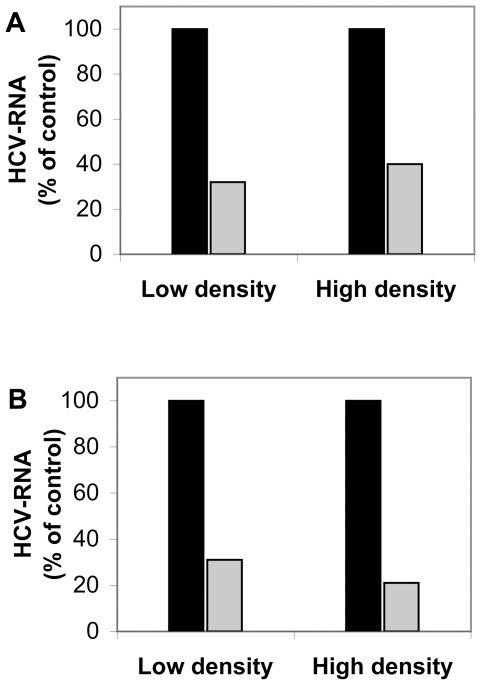
Cell infection with low- and high-density virus populations from iodixanol gradients in the presence or absence of LPL. The pooled peak fractions of the low- and high-density virus populations obtained after centrifugation through iodixanol gradients of JFH1 grown in cell culture (A) and serum samples from the m-JFH1 chimeric mouse model (B) (both gradients are shown in Figure 2) were used to infect cells in the presence or absence of 1 µg/ml LPL, as described in the Materials and Methods section. Cells were grown for 48 h at 37°C and HCV RNA was extracted and quantified by RT-qPCR. The results were normalized with respect to the cellular gene GAPDH, with the GAPDH Control Kit. The data are expressed as the amount of HCV RNA detected in cells infected with the pooled fractions from the two major virus populations in the presence of LPL as compared with the amount of HCV RNA in cells infected with the same fractions in the absence of LPL, expressed as a percentage.

Despite all these differences in density, LPL inhibited the infection of cells by all virus populations, regardless of whether the viruses were produced *in vitro* or *in vivo* (Figure 3A and B).

### Influence of LPL on the lipoprotein metabolism of Huh7.5 cells

LPL has two physiological roles. Firstly, it displays lipolytic activity, hydrolyzing the primary ester linkages in triacylglycerols from chylomicrons and VLDL, producing free fatty acids and monoacylglycerols. Secondly, LPL promotes the cellular uptake of chylomicron remnants, and cholesterol-rich lipoproteins through its role as a bridge between lipoproteins and HSPG at the hepatocyte surface. This function is independent of the catalytic activity of LPL.

We investigated the mechanisms by which LPL inhibited HCV infection, by analyzing the influence of LPL on VLDL hydrolysis and uptake by Huh7.5 cells under the experimental conditions used for HCV infection. Huh7.5 cells were washed with FCS-free medium and incubated with LPL on ice. Purified VLDL were then added and, after 30 min, the cells were transferred to 37°C and incubated for a further 2 h.

**Figure 4:**
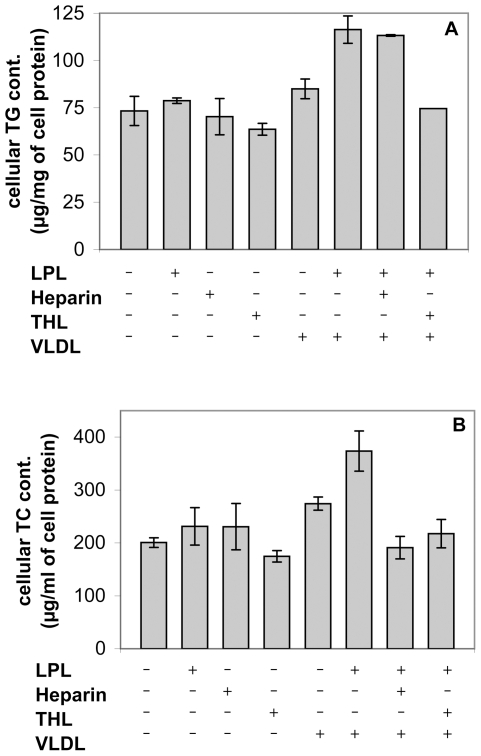
Analysis of the influence of LPL on VLDL hydrolysis and uptake by Huh 7.5 cells. Huh 7.5 cells were washed with medium without FCS and pre-incubated with LPL on ice. Purified VLDL were then added and, after 30 min, cells were transferred to 37°C and incubated for a further 2 h. The effect of LPL on VLDL uptake was shown by the increase in the amounts of both total triglycerides (TG) associated with cells in the presence of LPL. Cellular levels of TC and TG were also assessed in the presence of LPL and THL or heparin. (A) Hydrolysis of VLDL by LPL increased cellular TG levels due to the intracellular accumulation of fatty acids. This process was inhibited by THL, but was insensitive to the action of heparin, as it did not involve “bridging” mechanisms. (B) LPL increased the levels of TC in the cells in the presence VLDL. The effect of LPL was abolished by adding either THL, an inhibitor of LPL enzyme activity, or heparin, which prevents LPL from associating with proteoglycans. Thus, both catalytic and bridging activities were involved.

The effect of LPL on VLDL was already observable after 2 h of incubation, as shown by increases in the levels of both triglycerides (TG) and total cholesterol (TC) associated with the cells in the presence of LPL (Figure 4). We used tetrahydrolipstatin (THL), a specific inhibitor of the catalytic activity of LPL, which blocks the active site of the enzyme [48], to assess the impact of the lipolytic function of LPL on lipoprotein uptake.

Hydrolysis of VLDL was illustrated by the increase in TG levels resulting from the formation of fatty acids and their intracellular accumulation (Figure 4A). This process was entirely dependent on lipolysis and was therefore inhibited by THL, but not sensitive to heparin.

By contrast, the increase in TC after the incubation of cells with LPL and VLDL showed that the uptake of lipoproteins required both lipolytic and bridging activities (Figure 4B). Indeed, the simultaneous addition of THL and LPL abolished the stimulatory effect of LPL on lipoprotein uptake, by blocking its catalytic activity. The stimulatory effect of LPL was also abolished by the addition of heparin, which prevented LPL from associating with proteoglycans, thus disabling its “bridging” activity.

These data confirmed that, under our experimental conditions, both enzymatic and bridging activities were involved in the cellular binding and/or uptake of VLDL and their remnants. Indeed, the LPL-induced increase in TC associated with these cells required lipolytic digestion and heparan sulfate for the “docking” of the enzyme during its bridging function.

### Mechanism of action of LPL on HCV infection

**Figure 5:**
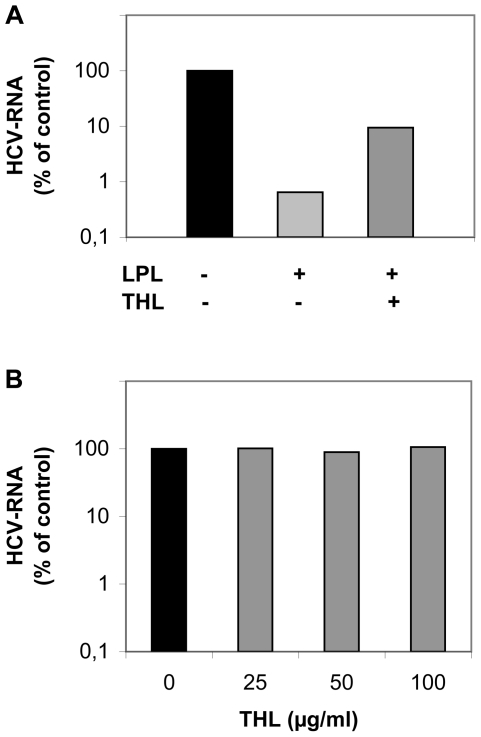
The inhibitory effect of LPL on HCVcc infection is only partly related to its catalytic activity. (A) Huh7.5 cells were pre-incubated with 1 µg/ml LPL at 4°C in the presence or absence of 50 µg/ml THL before infection with JFH1. The infected cells were grown for 24 h and HCV RNA was then extracted and quantified by RT-qPCR. (B) THL does not influence HCV replication. Huh7.5 cells were pre-incubated with indicated concentrations of THL before cell infection with JFH-1, as for experiments with LPL. THL was maintained in the medium for 24 h post infection. HCV RNA was then extracted and quantified by RT-qPCR. Results are expressed as a percent of RNA as compared with control cells infected in the absence of LPL and THL.

LPL has two physiological roles, either of which may affect virus infectivity. Its enzymatic activity might induce changes in the lipid composition and structure of the virus particles, whereas its “bridging” function might play a role in the inhibitory process, either binding the virus tightly to the cell surface or directing it to the abortive infection pathway. We investigated which of these LPL functions was determinant for the inhibition of HCV infection, by performing experiments with THL, blocking the catalytic activity of LPL. The inhibitory effect of LPL on HCVcc infection was only partially abrogated by THL (Figure 5A). In the absence of THL, LPL inhibited viral infection by two orders of magnitude, whereas, in the presence of THL, LPL also decreased infection levels, but only by one order of magnitude. We confirmed that THL alone had no influence on viral replication capacity, by measuring intracellular HCV RNA levels, 24 h after infection (Figure 5B).

**Figure 6:**
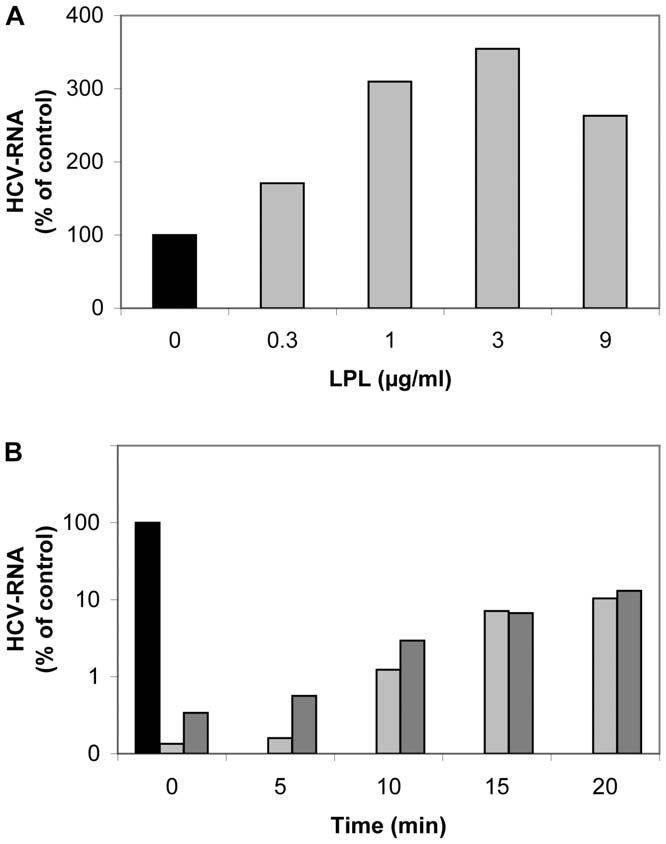
LPL affects HCV attachment and early stages of the virus cell cycle. (A) Effect of LPL on virus attachment to Huh7.5 cells. Huh7.5 cells were pre-incubated with various concentrations of LPL (1–9 µg/ml) for 30 min at 4°C. An aliquot of cell culture supernatant containing JFH-1 was incubated with LPL-pretreated Huh7.5 cells for 30 min at 4°C. The cells were washed and the RNA associated with them was extracted. HCV RNA was quantified by RT-qPCR. (B) Effect of LPL on early steps of HCV infection. JFH-1 was first adsorbed onto Huh7.5 cells by incubation for 45 min at 4°C. Cells were washed with cold medium to remove any unbound virus. Complete medium, warmed to 37°C, was then added and incubated with the cells at 37°C. LPL was added to a concentration of 1 µg/ml at various time points (0, 5, 10, 15 or 20 min) after the transfer of cells to 37°C, with or without the addition of 50 µg/ml THL to block its enzymatic activity. Cells were grown for 24 h. RNA was then extracted and HCV RNA was quantified by RT-qPCR. Results are expressed as a percent of RNA as compared with control cells infected in the absence of LPL.

These data indicated that the changes in the lipid composition of the virus particles induced by catalytically active LPL were only partly responsible for the inhibitory effect of the enzyme on viral infectivity and suggested a predominant role for the second, structure-bridging function of LPL. Indeed, exogenously added LPL enhanced the binding of HCVcc to Huh7.5 cells (at 4°C), as shown by the increase in HCV-RNA associated with cells (Figure 6A). This increase was maximal for a concentration of 1–3 µg/ml LPL. Higher concentrations of LPL did not increase virus binding further and were instead toxic to cells.

The enzyme influenced apparently virus cell entry, as infection levels were significantly lower following the addition of LPL during the first 20 min after infection (Figure 6B), and no significant inhibition was observed when the enzyme was added at later time points with the maintenance of its levels during cell culture. These results support the notion that LPL influences virus binding to the cell membrane and the early stages of HCV cell entry.

We therefore hypothesized that LPL could either block the virus at the cell surface, or internalize it by the non productive pathway, leading to abortive infection. To distinguish between these two possibilities, we followed the internalization of viral particles into Huh7.5 cells during first 20 min of infection, by electron microscopy. For these studies, virus particles produced in Huh7.5 cells (JFH-1 strain) and secreted into the cell supernatant were highly concentrated by ultracentrifugation through a sucrose cushion, as described in the Materials and Methods section. Huh7.5 cells were infected with the virus in the presence or absence of LPL and analyzed at the binding stage (T0) and after 5 min (T5), 10 min (T10) and 20 min (T20) of incubation at 37°C.

In the absence of immunogold labeling of viral proteins, it was not possible to localize viral structures to either the vicinity of the cells or within cells. These observations are consistent with the lipoprotein structure of the virus. We therefore performed immunoelectron microscopy (IEM) on fixed cells after various periods of incubation with HCV, using antibodies directed against HCV E2 or core proteins, followed by immunogold labeling. We used this experimental strategy to investigate viral cell entry in the presence and absence of LPL.

**Figure 7:**
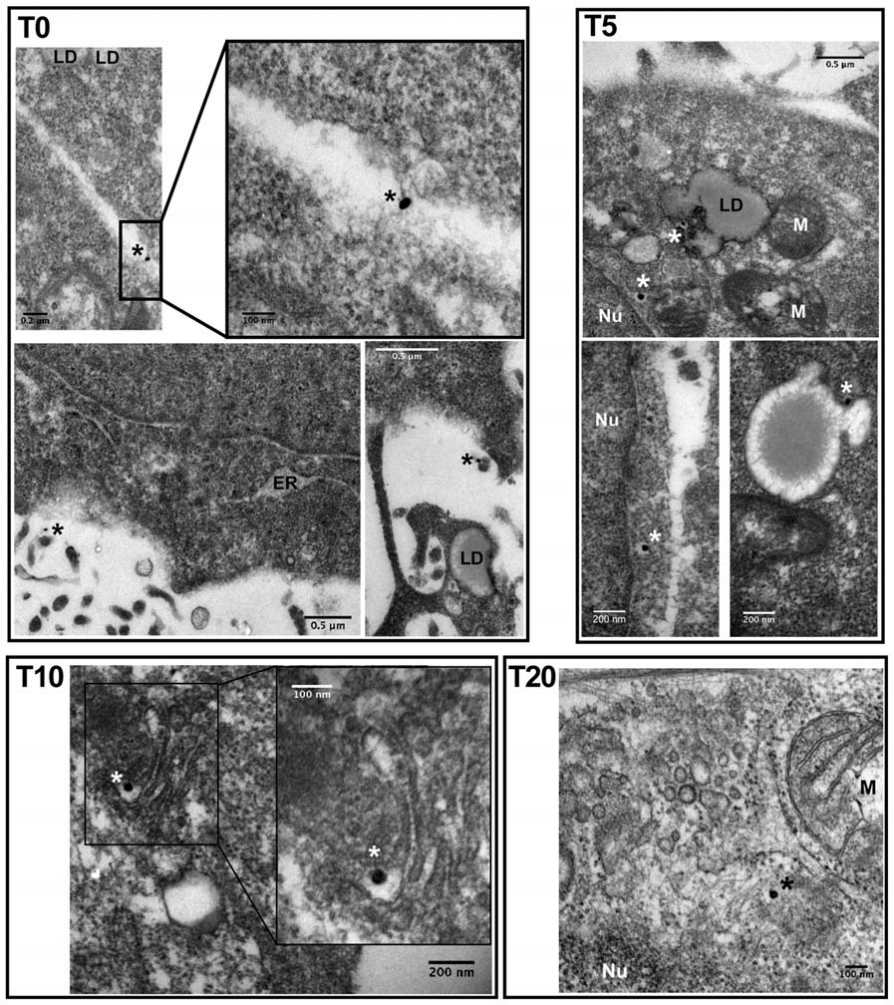
Immunoelectron microscopy of the infection of Huh 7.5 cells with HCV in the absence of LPL. The viral preparation (JFH-1) was concentrated by centrifugation through a sucrose cushion and incubated with Huh7.5 cells at 4°C (T0), before transfer to 37°C and incubation for a further 5 (T5), 10 (T10), 15 (T15) or 20 (T20) min. Cells collected at all these time points were washed, fixed and stained with monoclonal antibodies, followed by secondary, colloidal gold-labeled anti-mouse IgG (see Materials and Methods section for details). T0 and T5, immunogold labeling of HCV E2 envelope glycoprotein with monoclonal AP-33 antibody; T10 and T20, immunogold labeling of HCV core protein with monoclonal ACAP-27 antibody. Asterisks indicate the presence of one silver-enhanced gold particle. ER, endoplasmic reticulum; LD, lipid droplet; M, mitochondrion; Nu, nucleus.

In the absence of LPL, at the initial attachment phase, HCV was visible only on the outside of the cell, mostly in contact with the hepatocyte plasma membrane (Figure 7, T0). HCV appeared in intracellular vesicles after attachment of the virus at 4°C, followed by 5 min of incubation at 37°C (T5). After longer periods of incubation (10–20 min) at 37°C, HCV progressed further into the cell and was seen in vesicles localized in the cell center (T10), close to lipid droplets, and eventually close to the nucleus (T20). We chose to label both the HCV E2 and HCV core proteins, reasoning that the signal for HCV E2 might be lost at later time points, during viral internalization. However, we noted that the structures labeled with antibodies against E2 or core antibodies were identical, at all time points. The identical distributions of core and E2 suggested that our electron microscopy analyses followed the internalization of complete virus particles.

In control experiments, in which detergent-free buffer was used for the preparation of material for IEM, only the E2 protein was detected and found to be located outside the cell. Thus, saponin treatment was required for the intracellular staining of virus particles, suggesting that this treatment exposed HCV core from complete virions, as previously reported [15], [49].

**Figure 8:**
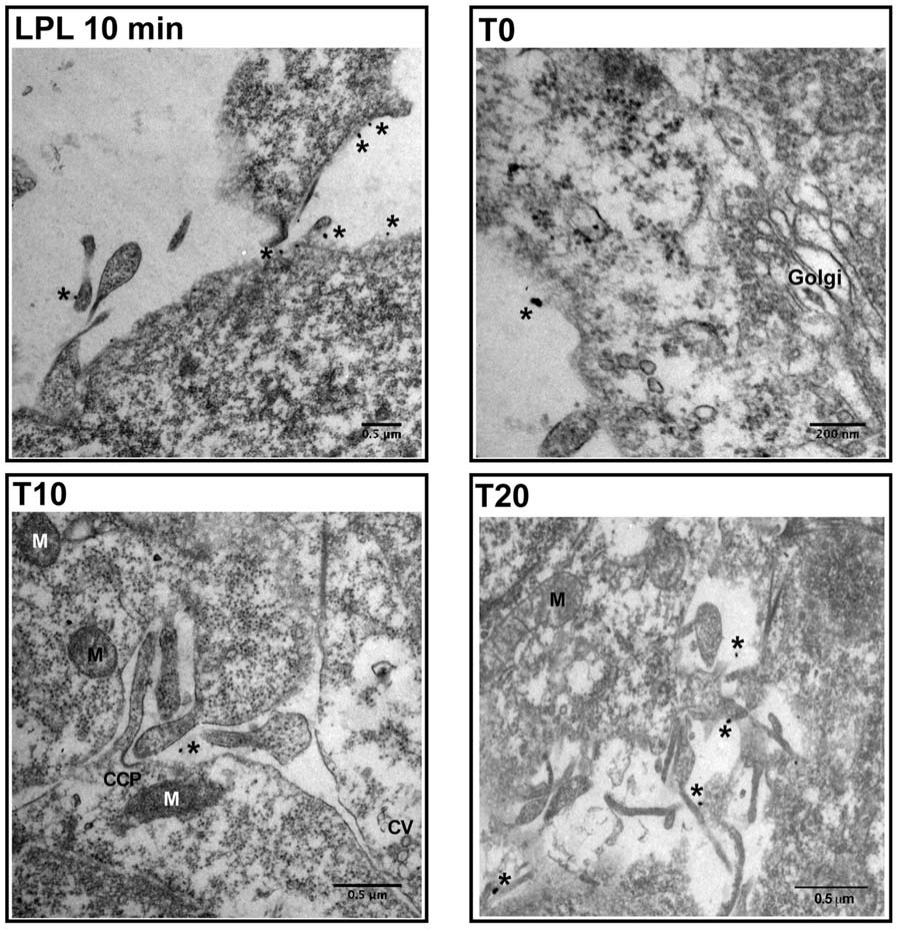
Immunoelectron microscopy of the infection of Huh 7.5 cells in the presence of LPL. Huh7.5 cells were pre-incubated with 1 µg/ml LPL, as described in Materials and Methods. The viral preparation, concentrated by centrifugation through a sucrose cushion, was incubated with cells at 4°C (T0), then transferred to 37°C and incubated for a further 5 (T5), 10 (T10), 15 (T15) or 20 (T20) min. Cells were washed, fixed and stained with anti-E2 (AP-33) or anti-core (ACAP-27) monoclonal antibodies, followed by secondary, colloidal gold-labeled anti-mouse IgG. LPL 10 min is a representative view of uninfected cells, pretreated with LPL at 4°C and subsequently for 10 min at 37°C, before immunogold labeling with anti-LPL antibodies and processing for TEM. T0, T10 and T20, show immunogold labeling with antibodies directed against HCV E2 (T0) and core protein (T10 and T20). Asterisks denote the presence of one silver-enhanced gold particle. CCP, clathrin-coated pit; CV, clathrin vesicle; M, mitochondrion.

In the presence of LPL, HCV was observed exclusively extracellularly, in close proximity to the plasma membrane, at all time points: T0, T10 and T20 (Figure 8). A very similar distribution was observed with anti-E2 and anti-core antibodies, indicating that, even at longer incubation times, complete HCV particles containing the virus nucleocapsid and envelope were still attached to the plasma membrane. Immunogold labeling of uninfected cells with an antibody against LPL was exclusively confined to the plasma membrane (Figure 8; LPL 10 min), suggesting that the enzyme also remained bound to the plasma membrane, or was degraded inside the cell. Uninfected cells incubated with the buffer used to dissolve LPL alone had a normal morphology and ultrastructure (data not shown).

We confirmed the specificity of the immunostaining procedure by performing several control experiments, including the labeling of infected or non infected cells in the absence of primary antibodies. No gold labeling was observed when the primary antibody was omitted or used on non infected cells. Staining with monoclonal antibodies against Rab-5 and Lamp-1 (instead of anti-E2 and anti-core) under the same conditions resulted in the specific labeling of early endosomes and late endosomes/lysosomes, respectively, with no labeling of any other structures (Figure S1).

**Figure 9:**
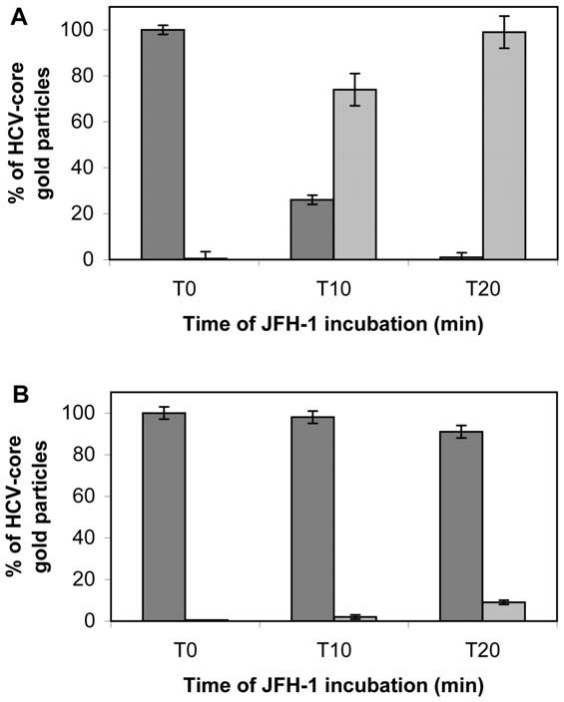
Quantitative analyses of immunoelectron microscopy. Cells were infected and processed as for Figures 7 and 8. HCV-bound gold particles after staining with anti-core antibodies were quantified in Huh7.5 cells infected in the absence (panel A) and presence (panel B) of LPL. For each condition and each time point indicated, 30 cells were analyzed, and the number of gold particles present outside (dark gray) and within (light gray) cells is reported. Data are expressed as means±SD.

We quantified the gold-labeled HCV core particles within and outside cells infected in the presence or absence of LPL. These analyses clearly demonstrated a progressive increase of the number of intracellular gold-labeled core particles (from T0 to T20 min post infection) with respect to the number of particles located outside the cell when infection was carried out in the absence of LPL (Figure 9A). By contrast, in the presence of LPL, most virus particles remained extracellular, at all time points tested (Figure 9B).

These results at the nanometric scale are consistent with an inhibitory effect of LPL during the early stages of HCV cell entry. Our observations suggest that LPL neither destroyed the virus particles nor induced their internalization into structures likely to direct the virus to abortive infection. Instead, LPL blocked HCV internalization, firmly binding the virus to the cell surface.

## Discussion

In this study we provide evidence that lipoprotein lipase, a key enzyme in lipoprotein metabolism, inhibits HCV infection. Our data show that exogenous LPL increases viral attachment to the cell surface, but strongly inhibits infection of the cell by HCV, by two mechanisms: lipolytic activity and “molecular bridging”, together leading to the retention of virus particles at the cell surface. We followed the early stages of HCV infection by immunoelectron microscopy and demonstrated that virus internalization into hepatic cells was blocked in the presence of LPL, which immobilized the virus at the cell surface.

Several studies have highlighted the role of lipoproteins and lipids in the HCV life cycle (see for review [22], [42]. Infectious HCV circulates in the serum of patients, in association with VLDL and LDL [13], [15], [17], which are integral components of HCV particles [16]. Indeed, the morphogenesis and secretion of infectious HCV follow the VLDL assembly pathway, with the secretion of VLDL-associated virions from infected cells [5], [8], [46]. Lipoproteins associated with virus particles render HCV infectious, as shown in studies *in vitro* [15], [17], [18], [50] and *in vivo* [14], [20], promote viral uptake by the lipoprotein receptors LDL-R and/or SR-BI/Cla1 [10], [17], [51], [52], [53] and protect the virus against neutralizing antibodies [53], which are therefore unable to control chronic HCV infection [54], [55].

Our research provides further evidence of the essential role of the lipoproteins associated with virus particles in HCV infectivity. In a previous study, we showed that exogenously added LPL increased the attachment of authentic HCV from the serum of patients to different types of cells, including hepatoma cell lines, by a “bridging” mechanism involving the binding of LPL to virus-associated lipoproteins and to HSPG at the cell surface [32]. Indeed, virus attachment to the cell surface via LPL required HSPG at the cell surface: LPL did not bind the virus to HPSG-deficient or heparinase-digested cells. This “molecular bridging” of HCV to hepatoma cells was mediated by the dimeric form of LPL and was analogous to the mechanism by which the liver takes up lipoproteins from the bloodstream [32]. Although LPL increased the binding of cell culture-produced HCVcc to the surface of Huh7.5 cells, we observed an inhibitory effect of LPL on HCVcc infection [32].

These observations led us to analyze further the impact of LPL on HCV infection. We show here that LPL inhibits cell infection with two strains of HCV, JFH-1 and J6/JFH-1, which replicate in hepatoma cells *in vitro*. The JFH-1 strain was derived from a cloned genotype 2a HCV RNA from a patient with fulminant hepatitis C [37], whereas J6/JFH-1 is a chimeric strain in which the genes encoding the structural proteins, p7 and NS2 are derived from another virus, also of the 2a genotype (HC-J6) [38]. The differences in the amino-acid sequences of the envelope proteins of these two strains may account for the differences in their interaction with lipoproteins, thereby affecting their overall physicochemical properties, including slightly different buoyant density [56]. Despite these potential differences, the infection of cells with these two HCVcc strains was equally inhibited by LPL.

The HCV particles circulating in patients' sera during natural infection have a wide range of buoyant densities, due to their association with lipoproteins [16]. In addition to the presence of mixtures of genomes of various sequences, virus particles from patient sera are associated with lipoproteins of different TRL and cholesterol contents, accounting for differences in infectivity and ability to escape the immune response.

Low-density HCV has been shown to be highly infectious in chimpanzees [14], [57] and in cell cultures *in vitro* [17]. The low-density HCV particles, LVPs [15], may represent different proportions of the total viral load in the serum [16]. Moreover, LVPs follow continuous dynamics, which depends on their production and transfer onto TRL in the circulation [58]. Thus, authentic HCV produced in the liver not only uses VLDL assembly, maturation and secretion pathway but also is subjected to the intra-vascular modeling of virus-associated lipoproteins [42], [58].

Huh7 cells, which serve as model currently used for studies of HCV infection have important defects in the cellular lipid metabolism and thus the spectrum of ApoB–containing lipoprotein particles assembled and secreted by these hepatoma cells does not resembles that produced *in vivo* or in cultured primary human hepatocytes [43], [44]. Indeed, this hepatoma cell line, like HepG2, secretes relatively dense, lipid-poor ApoB lipoproteins, unlike the buoyant VLDL secreted *in vivo* by the human liver [45], [59]. Consequently, most of the secreted ApoB lipoproteins have biophysical properties similar to those of LDL particles, as only a small percentage of the ApoB produced is fully lipidated and secreted as mature VLDL. Deficiencies in the transfer of lipids to the nascent ApoB in hepatoma cell lines leads to degradation of most of the Apo B produced [45].

The particular lipoprotein composition of virus particles produced in the *in vitro* model may render them more sensitive to LPL than natural virus particles. We therefore investigated whether LPL could also inhibit cell infection with the same two virus strains produced under more natural conditions, in primary human hepatocytes transplanted into chimeric uPA-SCID mice with normal lipoprotein metabolism. The humanization of lipoprotein profiles in this mouse model is associated with HCV infection success [36]. We show that LPL inhibits cell infection with the viruses produced in cultured Huh7.5 cells or in chimeric uPA-SCID mice into which primary human hepatocytes are grafted. The viruses produced *in vitro* and *in vivo* were found to be equally sensitive to LPL.

We also analyzed the physicochemical characteristics of viruses produced *in vivo* and *in vitro,* to identify the virus fraction sensitive to LPL. By ultracentrifugation through iodixanol gradients, we separated both HCVcc strains produced in Huh 7.5 cells, into essentially two virus populations, as determined by the distribution of HCV core antigen. Both these virus populations colocalized with ApoB and cholesterol and their densities indicated that they were associated with lipoproteins. The low-density viral population was about 10 times more infectious than the higher density population for viruses produced *in vitro* and about 15 times for viruses produced *in vivo*, despite higher core antigen and HCV RNA content in high density population. These observations are consistent with previous findings [46] indicating that the HCV RNA peak for HCVcc strains does not coincide with virus infectivity and that RNA is present in a large excess with respect to the number of infectious virus particles in higher-density fractions. These probably encapsidated particles contain HCV RNA and core protein but fewer of the virus-associated lipoproteins required for virus infectivity. In fractionated mouse sera the high-density viral peak also contained most of the HCV RNA and core antigen but had a lower density than the corresponding peaks for virus strains produced *in vitro*. This virus population was about three times more infectious than the corresponding peak in the inoculum.

These findings are consistent with previous observations that viruses produced *in vivo* or in cultured primary human hepatocytes are of lower density than those produced in hepatoma cell culture [20], [46], [60] and support the notion that the infectivity of virus particles is inversely correlated with their density and thus depends on the lipoprotein composition of the virus. Both HCV produced *in vitro* and *in vivo* are associated with ApoB-lipoproteins and contain ApoE, and both apolipoproteins are required for virus infectivity [5], [7], [8], [9], [15], [16], [50], [61], [62]. Moreover, mature virions have high levels of cholesterol and sphingolipid, the ratio of which is crucial for virus stability: the depletion of cholesterol and the hydrolysis of sphingolipids decrease virus internalization and infectivity [18], [19]. In addition, glycosaminoglycans and lipoprotein receptors play essential role in HCV cell entry and initiation of infection and thus lipoprotein composition of virus particles is determinant for virus infectivity. Indeed, three of the major molecules mediating HCV cells entry SR-BI, LDL-R and HSPG interacts with virus associated lipids and lipoproteins [22].

In our study, LPL inhibited infection with “low-density” and “high-density” viral populations isolated from cell supernatants or from “humanized” mouse sera. This observation suggests that the density of the virus, and thus the biochemical composition of the lipoproteins associated with virus particles, does not significantly influence the effect of LPL on HCV infectivity. Indeed, LPL also interacts *in vivo* with various ApoB-containing lipoproteins, such as LDL, VLDL, chylomicrons and their remnants, with their diverse lipid compositions and different biophysical characteristics [24].

LPL essentially has two biological functions [23], [26], [28], [30], [63]. First, LPL hydrolyzes the triglycerides in TRL, such as chylomicrons and VLDL, providing free fatty acids for internalization, via the CD36 receptor, into cells in peripheral tissues [23], [30]. Lipolysis is activated by ApoC2, an essential cofactor of LPL, whereas ApoC3 inhibits this process [64]. LPL is functional in a dimeric form: LPL dimers are tethered to HSPG or GPIHBP1 at the endothelial surface. GPIHBP1 has recently been identified as a key platform for the LPL-mediated lipolysis of TRL on the microvascular endothelium [65].

In addition to hydrolyzing TRL on the microvascular endothelium, LPL targets lipoproteins and their remnants to the liver and mediates their uptake by heptocytes. Indeed, the liver is a major organ for the clearance of ApoB-containing and ApoE-enriched lipoproteins and their remnants [30], [63]. In particular, the hepatocyte HSPG chains (mainly syndecan-1) binding the enzyme display a much stronger ligand affinity than HSPG in other tissues [27], [30]. This explains why the injection of exogenous LPL results in the rapid hepatic clearance of lipoproteins [24]. The hepatic uptake of chylomicrons and VLDL from the bloodstream, via LPL, involves a second LPL function, known as “molecular bridging”, in which a bridge is formed between the TRL particle and HSPG at the cell surface. HSPG can mediate subsequent lipoprotein internalization alone [27], [29], [30], [66] or in concert with the lipoprotein receptors LRP, LDL-R, [23], [24], [26] and SR-BI [31].

We performed a series of control experiments to assess the impact of LPL on lipoprotein metabolism in hepatoma cells. We found that, under our experimental conditions, the enzyme increased the cellular uptake of both total cholesterol and triglycerides from VLDL substrate, and that the mechanisms of uptake involved were different (Figure 4). Indeed, the uptake of VLDL (and their remnants) involved both lipolysis and bridging mechanisms, whereas triglyceride uptake was dependent exclusively on the enzymatic activity of LPL.

Thus, the inhibitory effect of LPL on HCV infection may be related either to its enzymatic activity, inducing changes in the composition of virus-associated lipoproteins, or to its structural, “bridging” function, independent of lipolysis.

It has been reported that LPL from *Pseudomonas aeruginosa* displays strong virolytic activity against HCV from patient sera [67]. However, we have previously shown that LPL from *Pseudomonas* sp. neither affects HCV binding to cells nor inhibits HCV infection [32]. Thus, the effect of LPL on HCV infectivity is specific to bovine LPL, which closely resembles the human enzyme. Our control experiments excluded the possibility of a direct virolytic effect of LPL on HCV: the infectious potential of JFH-1 was not significantly decreased by incubation of the virus preparation with 1 µg/ml LPL *in vitro* for 4 h at 37°C before infection [32]). In another study, the *in vitro* digestion of HCVcc with much higher concentrations of bovine LPL (up to 500 µg/ml) induced changes in virus composition and direct inactivation of the virus [68]. We used a low LPL concentration (1 µg/ml), similar to the physiological levels currently used in lipoprotein metabolism studies. This concentration gave the significant enhancement of virus binding to cells and was not toxic. Under our experimental conditions, the enzyme was prebound to cells at 4°C and its levels were maintained during virus infection. In these conditions, LPL decreased HCV infection levels by two levels of magnitude.

Our observations suggest that the enzymatic activity of LPL against virus-associated lipids was only partly responsible for the inhibition of HCV infection, because enzymatically inactive LPL decreased infection levels by a factor of 10. Indeed, the effect of LPL on HCV infectivity was only partly abrogated by THL, a specific lipase inhibitor that also abolished the effect of LPL on VLDL uptake into Huh7 cells. THL is an active site inhibitor that binds to LPL covalently to form a stable complex, inducing tetramerization of the enzyme [48]. THL does not influence LPL binding to HSPG, because the enzyme-inhibitor complex has the same affinity for heparin as active LPL, and facilitates hepatic TRL binding and clearance independently of its catalytic function [48], [69]. As THL only partly (by one order of magnitude) decreased the inhibitory effect of LPL, we hypothesized that the bridging/binding activity of LPL was involved in the inhibition of HCV infection.

Apparently, there is no correlation between the bridging function and lipolytic activity. Indeed, naturally occurring enzymatically inactive genetic variants of human LPL, display normal bridging function [70]. Consistent with this notion, the anti-LPL antibody 5D2, which blocks both functions of the enzyme, completely abolished the effect of LPL [32].

Our immunoelectron microscopy studies made it possible to follow, for the first time, the early steps of Huh7.5 cell infection. We used immune labeling with both anti-E2 and anti-core antibodies and colloidal gold-labeled anti-mouse IgG. In the absence of LPL, the virions were detected within cells as soon as 5 min after infection. Most of the virus particles were detected within cells 20 min after infection.

Identical patterns of staining were obtained with the anti-E2 and anti-core antibodies, consistent with the internalization of complete HCV virions. Nevertheless when detergent-free buffer was used, only extracellular viral particles were observed, and these particles were stained with anti-E2, but not with anti-core antibodies. Thus HCV core was probably exposed due to the detergent treatment, consistent with the pre-embedding strategy used for IEM, and with earlier reports showing that core particles can be detected with anti-core antibody after the delipidation of HCV virions with detergents [15], [49].

When HCV infection was carried out in the presence of LPL, the virus particles were not detected inside the cells at any time point, with either anti-E2 or anti-core antibodies, whereas both LPL and virus particles were detected at the cell surface. Overall, our findings clearly indicate that LPL inhibits HCV internalization by immobilizing virus particles at the cell surface.

Our observations suggest that HCV internalization might be rapid, whereas *in vitro* infection can be neutralized by anti-CD81 antibodies even 30 min after virus binding [71], [72]. However, it has recently been shown that virus particles of different densities may initiate infections at different rates [73]. Thus, the HCV internalization observed in our study by immunelectron microscopy may correspond to cell infection with one of the virus populations present in our heterogeneous inoculum, reflecting the initial phase of a much longer infection process.

The molecular mechanisms underlying the tight binding of viruses to the cell surface in the presence of LPL require further investigation. The lipolytic activity of LPL does not destroy the virus structure, but LPL may (i) cause the remodeling of lipoproteins associated with the virus, resulting in the formation of smaller and denser lipoproteins, (ii) change the balance between ApoB and ApoC's in HCV particles, this ratio being determinant for virus cell entry, (iii) induce conformational changes in ApoB, and/or ApoE, thereby affecting their reactivity with lipoprotein receptors [30], [62], [74], [75]. These changes might enhance the interaction of virus particles with cells and block their internalisation.

LPL might also increase virus attachment to hepatocyte HSPG, which is considered to be the HCV-attachment factor at the human hepatocyte membrane [76], [77]. LDL and other ApoB-containing lipoproteins bind weakly to vascular proteoglycans, but much more tightly via their lipids to proteoglycan -bound LPL. The non catalytic, bridging function of LPL therefore facilitates their retention in the vessel wall by extracellular matrix proteoglycans [78], [79], [80]. HCV retention at the surface of hepatic cells may thus be governed by similar mechanisms: direct virus attachment to HSPG, followed by a shift to much stronger virus binding to HSPG via LPL-bridging.

Collectively, our findings indicate that LPL inhibits HCV infection by two mechanisms: lipolytic modification of the TRL associated with virus particles and a predominant effect of the bridging function, which may act in parallel with lipolysis. Together, these two functions of LPL facilitate the retention of the virus at the cell surface, significantly decreasing infection levels. Thus, molecules targeting the lipoproteins associated with virus particles could affect HCV infectivity and, as such, could have implications for future therapeutic approaches based on the inhibition of HCV infection. Indeed, HCV-lipid interactions may be attractive targets for the development of antiviral drugs, because the targeting of essential host cell factors could limit the development of escape mutations effective against drugs directly targeting virus components [42].

## Supporting Information

Figure S1 **Immunoelectron microscopy of uninfected Huh7.5 cells stained with Rab5 or LAMP-1 antibodies.** Similar procedure to that described in Materials and Methods was applied to the immuno-gold labeling of Rab5, a marker of early endosomes and LAMP-1 as a marker of late endosomes and lysosomes. Asterisks denote vesicles positive for Rab5. White tick denotes a characteristic lysosomal localization of LAMP-1. D, desmosome; KF, keratin fibers.
(TIF)
