# Supplementary figures and images for: Lipoprotein Lipase Inhibits Hepatitis C Virus (HCV) Infection by Blocking Virus Cell Entry
Source: PLoS One. 2011 Oct 21;6(10):e26637. doi: 10.1371/journal.pone.0026637 (PMC3198807; doi:10.1371/journal.pone.0026637)

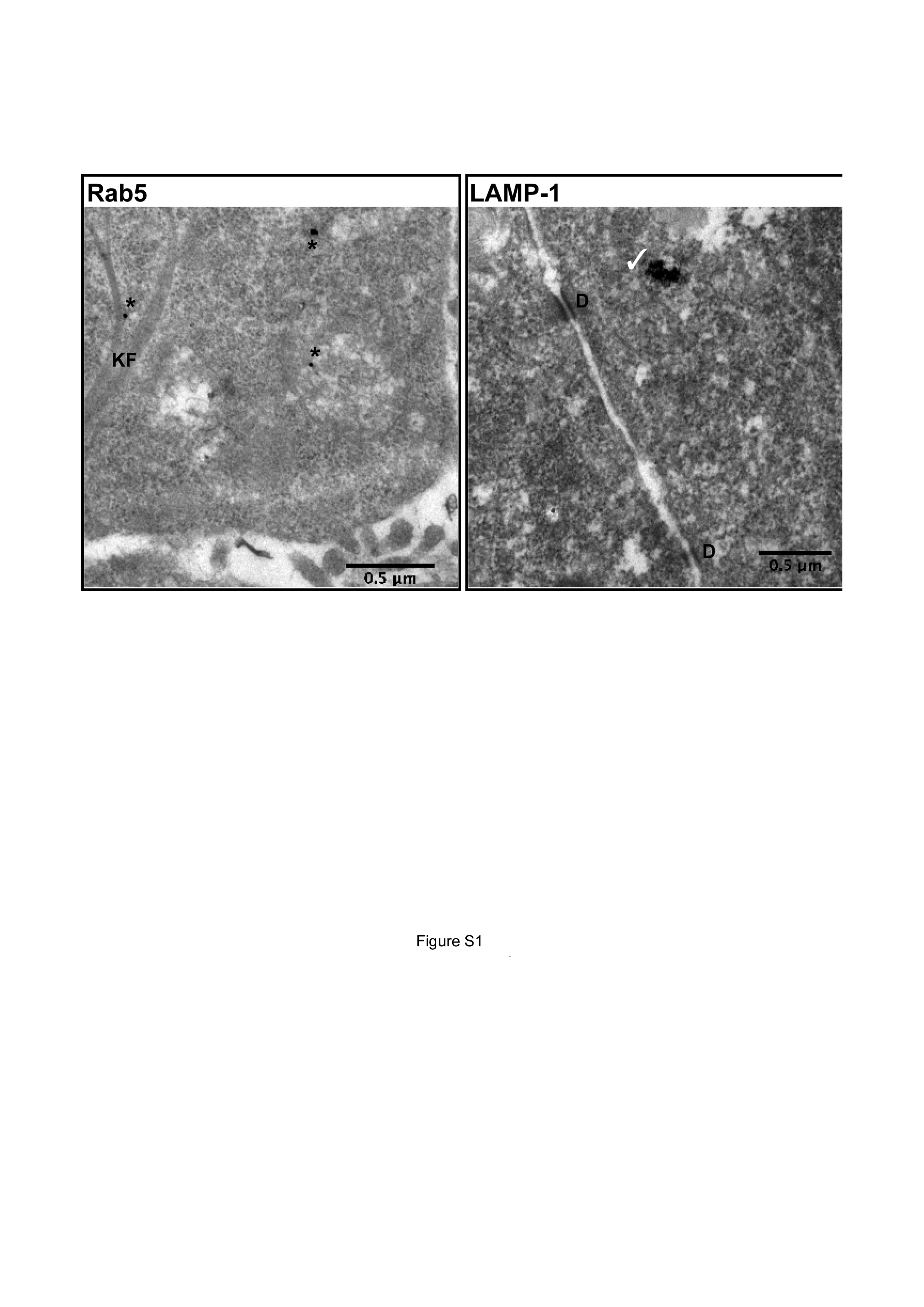

Supplement: Figure S1 — Immunoelectron microscopy of uninfected Huh7.5 cells stained with Rab5 or LAMP-1 antibodies. Similar procedure to that described in Materials and Methods was applied to the immuno-gold labeling of Rab5, a marker of early endosomes and LAMP-1 as a marker of late endosomes and lysosomes. Asterisks denote vesicles positive for Rab5. White tick denotes a characteristic lysosomal localization of LAMP-1. D, desmosome; KF, keratin fibers. (TIF) [file pone.0026637.s001.tif]
